# Home cage measures of Alzheimer's disease in the rTg4510 mouse model

**DOI:** 10.1111/gbb.12795

**Published:** 2022-01-19

**Authors:** Amanda J. Barabas, Lindsey A. Robbins, Brianna N. Gaskill

**Affiliations:** ^1^ Department of Animal Science Purdue University West Lafayette Indiana USA

**Keywords:** Alzheimer's disease, animal behavior, back‐translation, ethology, food grinding, mouse model, olfaction, preclinical model, rTg4510, tauopathy

## Abstract

Alzheimer's disease affects an array of activities in patients' daily lives but measures other than memory are rarely evaluated in animal models. Home cage behavior, however, may provide an opportunity to back translate a variety of measures seen in human disease progression to animal models, providing external and face validity. The aim of this study was to evaluate if home cage measures could indicate disease in the rTg4510 mouse model. We hypothesized that sleep, nesting, and smell discrimination would be altered in mutant mice. Thirty‐two transgenic mice were used in a Latin square design of four genotypes x both sexes x two diets. Half the mice received a doxycycline diet to suppress tauopathy and evaluate tau severity on various measures. At 8‐, 12‐, and 16‐weeks old, 24 h activity/sleep patterns, nest complexity, and odor discrimination were measured. After 16‐weeks, tau concentration in the brain was quantified. Mutant mice had increased tau concentration in brain tissue, but it was reduced by the doxycycline diet. However, only nest complexity was different between mutant mice and controls. Overall, tauopathy in rTg4510 mice does seem to affect these commonly observed symptoms in human patients. However, while running this study, a report showed that the rTg4510 mutant phenotype is not caused by the mutation itself, but confounding factors from transgene insertion. Combined with report findings and our data, the rTg4510 model may not be an ideal model for all aspects of human Alzheimer's disease.

## Graphical abstract

Data from mouse models will be more translatable if species relevant measures are recorded. Home cage behavior of the rTg4510 model of Alzheimer's disease was compared to wild type and hemizygous controls. Mutants only differed in nesting ability, a natural behavior that represents daily task performance in humans.

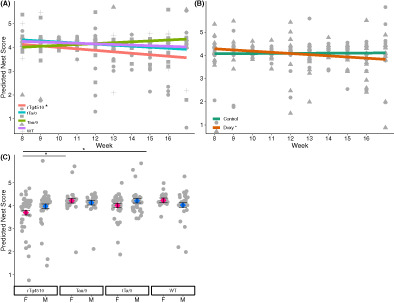

## 1 INTRODUCTION

Starting in the early stages of Alzheimer's disease (AD), short‐term memory deficits are a defining symptomology that leads to progressive difficulty in performing daily tasks (i.e., making the bed, cooking meals).^1^ However, this is not the only impairment found in AD patients. Neuropsychiatric symptoms also emerge as AD progresses including hyperactivity, sleep disruption, and abnormal personality changes.^1^, ^2^ Progressive olfactory deficits are also well documented in human patients: olfactory dysfunction combined with the lack of dysfunction awareness in patients with mild cognitive impairment can differentiate which patients will go on to develop AD.^3^, ^4^ Further, it has been argued that olfactory tests can better distinguish between AD and affective disorders than those that only assess mental state.^4^

Although not the only indicator of disease progression, memory impairment has historically been the earliest indicator of the disease, so animal models of AD are typically assessed for spatial memory deficits using the Morris Water Maze (MWM).^5^ Originally designed for rats, the MWM is used in both rat and mouse models.^6^ This is problematic for mouse models, who, unlike rats, find water to be aversive. Mice find training for the MWM stressful, which potentially impacts the quality of data from real trials.^7^ Even basic handling required to move mice from their cage to a testing arena is stressful^8^, ^9^ and how a mouse is handled can influence anxiety and olfactory discrimination outcomes.^10^, ^11^ Even these basic human‐animal interactions have the potential to introduce a large amount of variability to behavioral data.^12^ Further, excess stress can result in hypothalamic pituitary adrenal axis driven responses that have wide‐reaching effects throughout the body and add unaccounted variation to research data.^13^, ^14^, ^15^ Given the astronomically low rate of translation seen in AD research,^16^ it may be time to utilize other measures of disease in mouse models, ideally those that can be back‐translated from measures used in human patients, to improve translation. Thus, we should ask questions that are both relevant to human health and can be answered by the model species' natural behaviors. An appropriate disease model should display impairments in measures that are broadly applicable (external validity) and equivalent to the human condition (face validity).^17^

Since a variety of neuropsychiatric symptoms are seen in AD, testing is best suited within the animal's home cage. In contrast to typical testing arenas, home cage behavioral measures are less stressful because the mice remain in a familiar environment, undergo less handling, and allow the researcher to record more subtle changes in a broader range of behaviors.^18^ The following AD measures directly back translate measures in human patients to mice, which can easily be recorded in the home cage or with low stress handling techniques.^19^ Olfactory deficits are early indicators of AD in humans^3^, ^4^ and have been observed in both amyloid‐β and tauopathy mouse models.^20^, ^21^ Sleep patterns are commonly disturbed in human patients^2^ and have been shown to differ in several murine models of AD compared to wild‐type mice.^22^, ^23^, ^24^ Nesting behaviors in mice, such as nest complexity score, reflect complex daily tasks that are lost in AD patients, and reduced scores are proven indicators of disease state and pain.^25^, ^26^ APP/PS1 and rTg4510 models have even been shown to build less complex nests than wild types.^27^, ^28^, ^29^, ^30^ Postmortem body composition could also be a low‐stress measure of AD. Low bone mineral density has been reported in a murine model of tauopathy^31^ and quantities of lean and fat tissue mass could be indicative of hyperactive behavior, typically seen in humans.^2^ Since behavior observation is a time‐intensive process, these postmortem measures could be more practical for researchers.

Both amyloid‐β plaques and neurofibrillary tangles from tauopathy are classic biomarkers of AD,^32^ but tau buildup is a better predictor of cognitive impairments,^33^ leading to more research with animal models of tauopathy. A common tauopathy model is the bi‐transgenic rTg4510 mouse. Mutants display characteristic tau tangles in the cortex by 4 months of age and in the hippocampus by 5.5 months of age, which can be reduced with administration of doxycycline.^34^ During behavior tests outside of the home cage, rTg4510 mutants show spatial memory, learning, and motor impairments.^35^, ^36^, ^37^, ^38^ Hyperactivity in an open field arena has also been documented in 2‐month‐old females^38^ and 4‐month‐old males,^35^ but when both sexes are simultaneously evaluated, mutant hyperactivity is not increased until 6 months of age.^36^ The only behavior assessment in a familiar environment shows that mutant mice display age‐progressive hyperactivity between 16 and 32 weeks old, which could be reduced with doxycycline administration.^39^

Although rTg4510 mice show deficits in common test batteries, home cage measures could better represent disease in this model. Further, sleep behavior has not been evaluated in this particular mouse model. While nesting behavior has been recorded in the rTg4510 mouse, very little material was given, which limits the complexity of the nest and may limit differences between mutants and controls.^27^, ^30^ Past work did not use a previously validated measure of nest building, which recommends at least 6 g of material be given for mice to potentially construct a complete dome nest.^40^ Additionally, while deficits in olfactory discrimination have been shown in the Tα1‐3RT model of tauopathy,^20^ it has not been examined in the rTg4510 model.

The goal of this study was to determine if home cage behaviors, which reflect a more diverse array of human AD symptoms, are impaired in rTg4510 mutant mice. A model that shows deficits in a broad range of behavioral measures that reflect the human condition would demonstrate face and external validity. When compared to controls, we predicted that mutants would display more disruptive sleep patterns, lower quality nests, and reduced olfactory discrimination. These effects would become more severe over time as mutant tauopathy increases in severity; however, administration of a doxycycline diet would reduce disease progression in affected genotypes.

## 2 METHODS

### 2.1 Animals and housing

All methods and procedures were approved by Purdue University's Institutional Animal Care and Use Committee (protocol #1811001822), but the protocol was not publicly registered in a preclinical database. Since AD is a progressive disease, mice were monitored daily for signs of pain and distress, and body weights were taken weekly. A 20% reduction in body weight was used as humane endpoint criteria, but no mice met this criterion.

This study used a total of 32129S6;FVB‐Tg(Camk2a‐tTA)1Mmay Tg(tet‐O‐MAPT* P301L) mice^34^ (rTg4510) from a colony owned by Eli Lilly and Co and managed at Taconic Biosciences (Indianapolis, IN). This sample size was estimated a‐priori using Mead's resource equation.^41^, ^42^ The following factors were used in our four genotype × two diet × two sex Latin square design. Within each genotype/diet combination, mice were equally divided across sex (*n* = 2). Mutant rTg4510 mice are a product of an F1 cross between a 129S line containing a tetracycline transactivator and an FVB line containing the human tau responder. Since neither gene is endogenous to mice, only one copy of each is inserted into the parental lines. A single copy of both transgenes is sufficient to produce tauopathy in mutant offspring: in brief, the inserted transactivator is required for the inserted human tau to be expressed. For more details, please refer to.^34^ rTg4510 mutants (rTg4510) were compared to the following littermate controls: wild‐type (WT), and hemizygous for either the transactivator (tTa/0) or the responder (Tau/0). Hemizygous mice were included to rule out single gene effects in rTg4510 phenotype after anecdotal observations of hyperactivity were reported in tTa/0 mice. A random number generator (random.org) was used to assign half of the mice in each genotype to a control diet (control; Envigo, Teklad 2016, Indianapolis, IN) or the control diet with 200 mg/kg doxycycline hyclate (doxy). The doxy diet was administered to regulate tauopathy as it turns off the transactivator, reducing tau expression^34^ and assess how tau severity affected outcome measures.

Mice arrived at 7 weeks of age and immediately received their assigned diet. Both diet and reverse osmosis water were available ad libitum throughout the study. Mice were individually housed in static cages (Ancare, Bellmore, NY) with aspen wood chip bedding (Envigo, Indianapolis, IN) and 10 g of virgin kraft crinkle paper nesting material (Fibercore, Cleveland, Ohio). Mice were handled using clear polycarbonate tubes (3 7/8“ long × 2” inside diameter; 1/8″ wall; BioServ, Flemington, NJ), which were kept in the cage with each mouse. They also received manzanita wood chew sticks (BioServ, Flemington, NJ) as enrichment. Cages were kept under a 12:12 light: dark cycle (lights on at 06:00) with relative humidity ranging 57.2 ± 3.1% and temperature ranging 22.6 ± 0.34°C. Cage changes were performed bi‐weekly.

Cage placement on the rack was randomized (random.org), such that each genotype was housed on each of the five rack shelves. However, it was not balanced across sex and diet as only seven cages maximum could fit on each shelf (the top four shelves held seven cages; the bottom held four). Each cage was numerically labeled, such that all researchers and care staff were blinded to genotype and sex. It was not possible to be blinded to diet as they were different colors.

Unless otherwise noted, all measures were taken at three‐time points during the study: 8, 12, and 16 weeks of age. A repeated measures procedure was used to document behavior as tauopathy progressed over time in mutants. Mice were given 7 days to acclimate to the facility before testing began at 8 weeks. We had to use an incomplete block design during testing, thus mice were rotated throughout the testing battery in groups of four (one of each genotype was represented and half of the cages represented each sex and diet). Test order was randomized across groups but kept constant within‐group across time points (Table S1). All researchers and care staff for this study were female. All testing was done within the housing room.

### 2.2 Repeated behavior measures

#### 2.2.1 Sleep patterns

Sleep patterns were recorded using previous methods.^43^ Briefly, a block of four mice were individually and randomly placed in one of four chambers (17.78 cm × 17.78 cm) in a noninvasive sleep monitoring apparatus (Signal Solutions, Lexington, KY, Figure S1). Piezoelectric mats underneath each chamber recorded vibrational movement for each mouse. Since the mats are sensitive to vibration, the apparatus was placed away from any equipment that may produce vibration (computer fans). Signals were processed with previously validated, specialized software (Mouse Rec Data Toolbox, Signal Solutions, Lexington, KY) to distinguish sleep and awake patterns. Each chamber had its own built‐in food hopper and water bottle, and mice were given nesting material and aspen from their home cage during testing. All mice were given 24 h to acclimate to the sleep apparatus before data collection. Sleep patterns were recorded for a full 24 h at each time point to calculate the proportion of time spent sleeping and the mean sleep bout length for each mouse at each time point.

#### 2.2.2 Nest scores

Nest quality scores were taken weekly throughout the study (10 weeks) at approximately 13:30 based on previous methods.^26^ Briefly, at evaluation, nests were divided into quadrants and graded on a scale of 0–5. Quadrant scores were averaged for an overall nest score. Scores of 0–1 indicate no manipulated nest structure; scores of 2 indicates gathered material with no walls; scores of 3 indicates a cup shape with walls lower than half of an imaginary sphere which would cover a mouse; scores of 4 indicates a nest with walls that reach half of an imaginary sphere; and scores of 5 indicates a nest with walls over half the height of a sphere, which may or may not be fully enclosed. All mice ultimately had 10 nest scores, except for one Tau/0 male on the doxy diet, whose cage flooded right before the first measurement.

#### 2.2.3 Olfactory discrimination

Procedures were based on previous methods.^44^ At each time point, mice were assessed for odor habituation/dishabituation. Briefly, mice were tested in seven odor trials. Trials 1–6 used the same odor, with which the mice should become familiar and display less interest with each progressing trial. The seventh trial used a novel odor which should renew the mice's interest. All odor combinations are listed in Table S2. Odor treatments were prepared by pipetting 5 μL of odor extract onto a clean piece of cotton gauze and placing them in clean tissue cartridges. The day before testing, an empty cartridge was placed within the mouse's home cage for acclimation. On trial days, mice were tested in their home cages, but food, water, and all enrichment were removed, so mice were given 1 h to acclimate to the change. Each trial lasted 30 s, with five‐minute intervals between trials. Researchers changed gloves between all trials to avoid odor cross‐contamination. All trials were recorded using infrared closed‐circuit television cameras (CCTV) (Sony, Tokyo, Japan) and GeoVision software (Taipei, Japan). At each time point, observers blinded to all treatments recorded the latency of each mouse to approach and the total time spent sniffing the odor cartridge during each trial. Only trials 1, 6, and 7 were used for analyses.

#### 2.2.4 Home cage behavior

At each time point, home cage behavior was monitored in video booths made by enclosing Metrorack shelves with white foam board (Office Depot, Boca Raton, FL) and installing CCTV cameras for overhead view. The camera model and software were the same as that used for the odor trials. During video observation, a custom cage setup was used. Holes were drilled into clear solid polysulfone lids (Alternative Designs, Siloam Springs, AR). Each cage had a hanging stainless steel feeder (Alternative Design, Siloam Springs, AR) and an externally mounted water bottle, so overhead behavior could be observed (Figure S2). Water was connected to the cage with medical‐grade silicone tubing. Flooding occurred in several cages throughout the experiment. This factor was tested in the final analyses, but it did not significantly impact behavior while in the video booths.

When the mice were 10 weeks of age, research staff anecdotally observed that several mice were performing an extreme amount of abnormal repetitive behaviors (ARB; often referred to as a stereotypy), such as circling or bar biting. ARBs in animals are considered a sign of poor animal welfare, often developing due to solitary or barren housing.^45^ As a prey species, laboratory mice are prone to developing stereotypies derived from escape attempts and can be more active as a consequence,^45^ which may contribute to the hyperactivity seen in this model. However, due to methodological limitations on past work, it is unknown whether hyperactivity is due to tauopathy or confounded with the development of ARBs. More detailed behavior observations could better distinguish disease indicators from ARBs caused by poor housing conditions. Therefore, ARBs were added to the list of behavior measures.

Mice were given 24 h to acclimate to the video booths and behavior was coded for the following 24 h. One–zero sampling for 1 min every 5 min was used to record when the mice were active and performing ARBs (Table 1). ARB definitions were taken from mousebehavior.org and included route tracing, looping, and jumping and were recorded if they occurred at least three times within 10 s. Route tracing and looping were primarily observed. Three main observers were blind to sex and genotype. For every mouse, at each time point, the proportion of active time in which an ARB occurred was determined by tallying the bins where each category was observed and dividing them by the number of bins in which the mouse was active. Inter‐rater reliability was acceptable for ARBs and excellent for general activity (Kappa >0.76 and 0.90 respectively).^46^

**TABLE 1:** Ethogram of behaviors for home cage observations. Descriptions were taken from mousebehavior.org

Category^a^	Behavior	Definition
Abnormal repetitive behavior^b^	Jumping	A repetitious upright motion towards the cage top. Sometimes when rearing, mice may jump up towards the cage lid. The mouse must do multiple jumps in one or multiple consecutive sessions.
	Looping (back flipping)	Before looping, a mouse will generally extend its body upward from the cage floor and rest its forepaws against a surface to brace itself. Then it will tilt its head back a couple times, while arching its back, to gain momentum and position for the back flip. The mouse completes a full loop. The loop can be cyclical or elliptical in shape. The hind paws of a mouse will often touch another surface before completing its landing onto the cage floor.
	Route tracing	A mouse will trace out an identical, repeated route around the cage lid or on the cage floor. This route can be in a circular pattern or another consistent, recognizable shape.
At feeder		Score this behavior if the mouse is seen at the feeder for at least 5 s. The mouse's nose must be seen in between the bars of the feeder.
Active		Score if the mouse is visible and alert (walking around cage, grooming, passively sitting) for more than 5 s.

^a^ Categories were scored using one–zero sampling over a 24‐hour period.

^b^ Scored if the mouse performed the same behavior three times within 10 s. Any abnormal behavior bout that began within the minute time bin was scored. If it continued past the end of the minute, it was still included.

When the mice were 10 weeks of age, research staff anecdotally noticed large visible quantities of orts, or powdered food waste, accumulating in some cage bottoms. Orts are indicative of abnormal grinding behavior in which the mice do not ingest food, but rather grind it into a powder. After the high amount of orts were noticed, it was decided to add time spent at the feeder to the home cage ethogram and quantify ort production for future husbandry purposes as well as to document this observation in the literature. During video observation, time spent at the feeder was recorded if the mice spent at least 5 s with their snout in between the feeder bars. For every mouse, at each time point, the proportion of active time in which feeder activity occurred was determined by tallying the bins where each category was observed and dividing them by the number of bins in which the mouse was active. Inter‐rater reliability was excellent (Kappa >0.90). Used bedding material was collected at cage change on weeks 12 and 16, representing the total amount of orts from the previous 2 weeks. Once collected, used bedding and orts were air‐dried in a 55°C oven (Animal Sciences Research and Education Center Purdue University, IN) overnight until the weight measurement was constant. Weight was considered constant if it did not change after three consecutive measurements, taken within 15 min of each other. The dried material was passed through a food sifter to separate the orts from bedding or fecal matter. Some cages experienced flooding during this study. Any orts present in a flooded cage between weeks 10–12 and 14–16 were collected and dried. Material that was added to the cage post‐flooding was also collected on the originally scheduled cage cleaning day. If orts were collected more than once per time point, due to a flooding incident, the orts collected over those time points were added together.

### 2.3 Tau concentration

After the 16‐week time point data was collected, mice were euthanized by prolonged exposure to CO_2_. Brain tissue was collected, connective tissue was removed, and the hemispheres were separated. Samples were flash frozen in 1.5 ml centrifuge tubes using dry ice and shipped for tau quantification at Eli Lilly and Co (Indianapolis, IN) using proprietary methods as done previously.^47^ Briefly, total protein content was determined using bicinchoninic acid assay (Pierce Biotechnology, Rockford, IL). Total tau levels were determined using an enzyme linked immunosorbent assay. Ultimately, the final concentration of tau (ng/mg total protein) was calculated.

### 2.4 Body composition

Mouse carcasses were frozen and taken to the Farm Animal Behavior Laboratory (West Lafayette, IN) for postmortem body composition analysis using the Norland pDEXA Saber densitometer (Norland Medical Systems, Inc.; DXA). Mice were thawed for 1 h before being scanned, ventral side down, using software version 1.1.1. Data were analyzed using software version 3.9.4 with histogram averaging set to automatic. The instrument was calibrated daily before use as recommended by the manufacturer. All DXA measurements started with a scout scan (resolution 1.01.0 mm; scan speed of 40 mm/s). The scout scan produced a screen image of the object with a superimposed “cursor box” that specified the areas for analysis. The curser box was adjusted to define the area of focus (the base of neck to base of tail) and measurements were confirmed using a ruler within 1 mm of the results given by the cursor box. All measurement scans were conducted with the resolution set to 0.5 mm and a scan speed of 8 mm/s. The total time necessary to scan an animal (scout plus measurement scan) ranged from 8 to 20 min, depending on the animal's size. All DXA scans and analyses were conducted by the same observer (SP) and were checked for reliability by a secondary observer (LR). DXA scans were analyzed to determine fat mass, lean tissue mass, and bone mineral density (BMD).

### 2.5 Statistics

#### 2.5.1 Repeated behavior measures

All repeated measures were analyzed in JMP Pro (version 14.0.0) using Restricted Maximum Likelihood general linear mixed models (GLMM) unless stated otherwise. The main effects of genotype, diet, sex, and time point were tested along with all two‐way interactions. Mouse ID nested in diet, sex, and genotype was included as a random factor and consequently had to be included as three‐way interaction in the models. We acknowledge that a larger sample size would be preferred to interpret three‐way interactions, and any significant three‐way interactions should be critically evaluated.

All assumptions were assessed post hoc using the residual by predicted plot and normal Q‐Q plot. Transformations were made when necessary. Any significant main effects were assessed with post‐hoc, Bonferroni corrected contrasts, or Tukey tests. Any non‐significant interactions were dropped from the final models. Any missing data points and model exceptions are listed below (Table S3 for additional information). No a priori criteria were established.

##### Sleep patterns

Sleep data is missing from four cages at week 16 due to equipment malfunction.

##### Nest scores

One nest score, from week 8, was excluded from a male Tau/0 mouse on the doxy diet due to a flooded cage. This model included location as a blocking factor, as some mice were in the sleep apparatus during observations. Since scores were recorded weekly, time point was included as a continuous variable.

##### Olfactory discrimination

Trial number was included as a main effect and in all interactions.

##### Home cage behavior

Two data points from week 12 were excluded as one small mouse figured out how to escape from its cage during the dark period and interacted with the cage on the same shelf, likely altering the data. The model for home cage activity included a covariate of ARB performance (as a proportion of active time). However, it was dropped due to insignificance. Welch's ANOVA was used to analyze ARB data for main effects of diet, sex, and genotype, since the data violated the homogeneity of variance assumption and transformations were not successful in meeting this assumption.

Equivalence tests were performed on the main behavioral measures analyzed with parametric tests: total proportion of time sleeping and total time sniffing the odor cartridge. This was done to determine whether the analyses had enough power to detect a biological difference.^48^ Equivalence ranges were taken from past studies on AD mouse models when possible.^20^, ^22^, ^23^, ^24^, ^44^, ^49^, ^50^

#### 2.5.2 Tau concentration and its influence on 16‐week measures

First, tau concentration was analyzed using a general linear model (GLM) in JMP Pro, testing main effects and interactions of genotype, diet, and sex. Two cages were excluded from tau models because of a suspected sample label switch during or after brain collection (Table S3).

Afterward, data from the 16‐week time point only were reanalyzed for effects of tau concentration, in addition to two‐ and three‐way interactions of genotype, diet, and sex. DXA data was also analyzed as a GLM with the same treatments. The models for lean and fat mass included the 16‐week proportion of active time in the home cage as a covariate. One cage was excluded from lean and fat mass models due to unreadable data (Table S3).

## 3 RESULTS

### 3.1 Repeated behavior measures

#### 3.1.1 Sleep patterns

Neither genotype nor any associated interaction had an impact on sleep measures (*p*'s > 0.05; Table S4). However, there was a main effect of sex on both proportion of time spent sleeping (*p* = 0.026) and mean sleep bout length (*p* = 0.005; Table S4). Males slept for more of the study period and had longer sleep bouts than females. For proportion of time slept, there was also a significant interaction between sex*diet*week (*p* = 0.015; Table S4). However, the only significant differences could not be explained by the treatments (Table S5). For mean sleep bout length, there was a significant main effect of week (*p* = 0.035; Table S4), but there were no significant post‐hoc differences. While differences between diet and genotype treatments were not large enough to be significant, equivalence tests were run to determine if a biological difference in the mean proportion of time slept could have been identified. A value of 0.0833 was determined a biologically meaningful difference based on average sleep proportion differences between mutant and WT mice of other AD models.^22^, ^23^, ^24^ A 0.0833 difference between diet treatments, in mutant mice only, unfortunately could not have been identified (t ratio = −1.47; P = 0.082). However, a 0.0833 difference could have been detected between mutant and wild‐type mice on control diets (t ratio = −2.83; P = 0.006). A least significant number calculation for the genotype*diet interaction showed that 16 total animals would be necessary to achieve 80% power with the observed effect size.

#### 3.1.2 Nest scores

**FIGURE 1:**
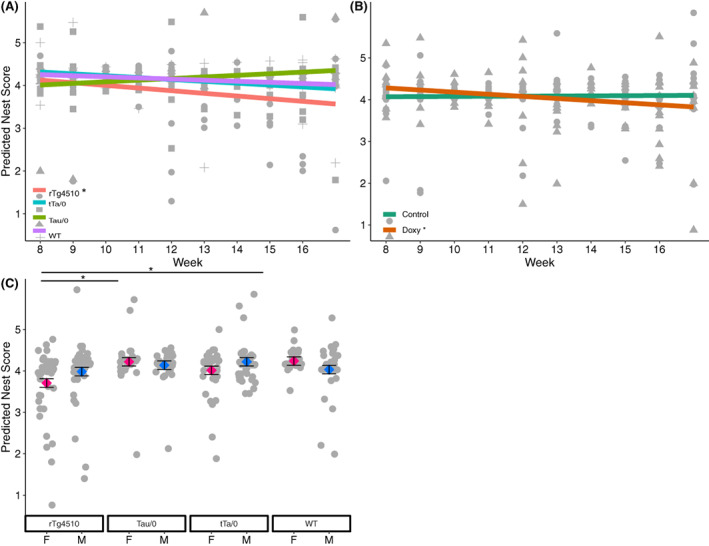
Weekly nest complexity in the rTg4510 mouse model. Nest scores were impacted by main effects of (A) genotype*week time point, (B) diet*week time point, and (C) sex*genotype (adj R^2^ = 0.38, *N* = 319). All predicted + residual data points are depicted as gray scatter. The predicted slope over time is depicted for each genotype (A) and diet (B). Those significantly different from zero are marked by * in the legends. Factor level LSM ± SE are presented in C. Significant differences in post hoc tests are depicted by *

Overall, nest complexity was high, regardless of genotype or diet. Average scores were all above 4 on the 5‐point scale (Table 2). Nest complexity was significantly impacted by the interaction of genotype*timepoint (*p* = 0.015), diet*timepoint (*p* = 0.018), sex*genotype (*p* = 0.038), and location (*p* < 0.001; Table S6). Bonferroni corrected custom tests showed that nest scores from rTg4510 mutants had a significant decrease over time, as well as scores from mice on the doxy diet (*α* = 0.05/6 = 0.008; *t* = −2.73, *p* = 0.007, Figure 1A; *t* = −3.12, *p* = 0.002, Figure 1B). Female rTg4510 mice had lower nest scores than female WT and female Tau/0 mice (Tukey: *p* < 0.05; Figure 1C). All mice had higher scores in their home cage compared to the sleep apparatus.

**TABLE 2:** Nest scores across genotype and diet based on the 5 point scale.^26^ Values represent treatment means ± SD

Genotype	Doxycycline diet	Control diet
rTg4510	4.45 ± 1.05	4.58 ± 0.85
tTa/0	4.89 ± 0.34	4.68 ± 0.74
Tau/0	4.85 ± 0.79	4.72 ± 0.94
WT	4.69 ± 0.91	4.92 ± 0.30

#### 3.1.3 Olfactory discrimination

**FIGURE 2:**
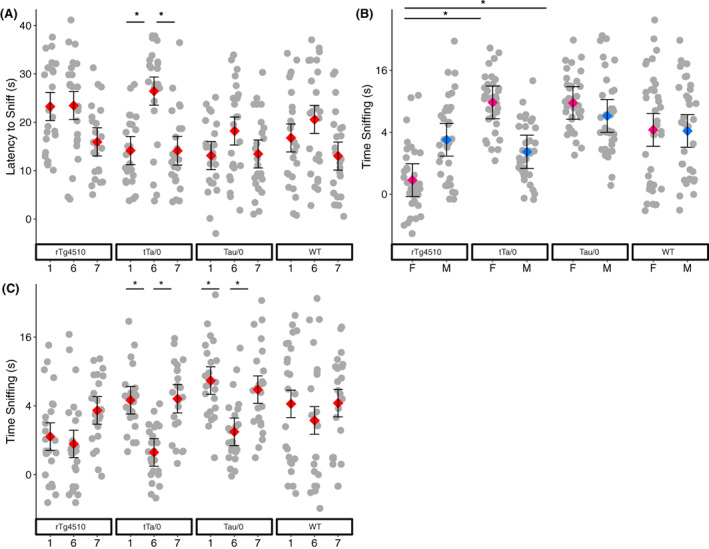
Odor discrimination testing in the rTg4510 mouse model. (A) Latency to sniff the odor cartridge was impacted by main effect of genotype*trial number (adj R^2^ = 0.36, *N* = 286). Total time sniffing the odor cartridge was impacted by main effects of (B) genotype*sex, and (C) genotype*trial (adj R^2^ = 0.34, *N* = 286). All factor level LSM ± SE are presented over a scatter of individual residual values. Y‐axes in B and C are presented on a square root back‐transformed scale. Significant differences in post hoc Tukey tests are depicted by *

The latency to approach the odor cartridge was impacted by trial number and the interactions of genotype* trial number (*p* = 0.019) and genotype*diet*week (*p* = 0.048; Table S7). In general, there was a longer latency to approach the odor in trial 6 compared to trials 1 and 7 (*p* < 0.001; Table S7). However, in tTa/0 mice, the pattern was strongest and was the only genotype with a significantly longer latency in trial 6 (Tukey: *p* < 0.05; Figure 2A). Tukey tests showed no significant differences between groups in the 3‐way interaction. Total time sniffing the odor was affected by trial number and the interactions of genotype*sex (*p* = 0.045), genotype* trial number (*p* = 0.034), and genotype*diet*week (*p* = 0.037; Table S7). In general, mice spent less time sniffing the odor in trial 6 compared to trials 1 and 7 (*p* < 0.001; Table S7). Female tTa/0 and female Tau/0 mice spent more time sniffing the odor than female rTg4510 mice (Tukey: *p* < 0.05; Figure 2B). In tTa/0 and Tau/0 mice, the pattern was strongest and were the only genotypes that spent a significantly shorter time the time spent sniffing the odor in trial 6 compared to trials 1 and 7 (Tukey: *p* < 0.05; Figure 2C). Tukey tests showed no significant differences between groups in the 3‐way interaction. Due to lack of significance, equivalence tests were again run to determine if a difference of at least 2.52 s could be detected. This value was considered a biologically meaningful difference based on differences between mutant and WT mice of other AD models.^20^, ^44^ A 2.52 s difference between diet treatments would have been detectable within mutant mice (t ratio = −4.66; *p* = 0.0001) and as well as a difference between mutant and wild‐type mice on the control diet (t ratio = −2.47; *p* = 0.0093).

#### 3.1.4 Home cage behavior

**FIGURE 3:**
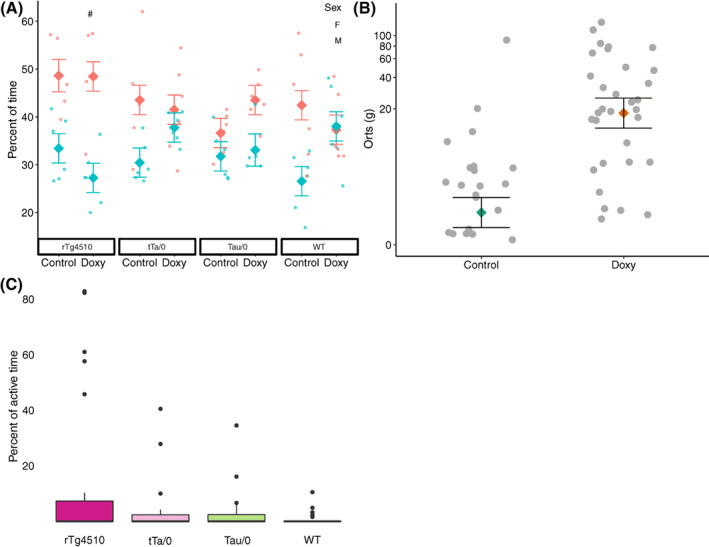
Home cage behavior in the rTg4510 mouse model. (A) Percent of time active in the home cage was impacted by the genotype*diet*sex interaction (adj R^2^ = 0.39, *N* = 94). Differences between sexes are depicted by a #. Sex main effect LSMs are depicted by horizontal lines. (B) The total amount of orts produced was impacted by diet (adj R^2^ = 0.60, *N* = 64). The percent of active time performing abnormal behavior (C) was influenced by genotype (N = 94). Factor level LSM ± SE are presented over a scatter of individual data point residual values in A and B while box and whisker plots of raw data are presented in C. Y axis in B is presented on a log10 back‐transformed scale

Activity in the home cage was affected by the interaction of sex*diet*genotype (*p* = 0.048; Table S8). Differences within genotype are highlighted here, with full pairwise contrasts shown in Table S9. Female rTg4510 mice on the doxy diet were more active than male rTg4510 on the doxy diet (Tukey: *p* < 0.05; Figure 3A).

Time spent at the feeder was impacted by the interaction of genotype*diet*week (*p* = 0.017; Table S8), but Tukey tests showed no differences between groups. Week influenced time at the feeder (*p* = 0.015; Table S8). Mice spent more time there at the 8‐week time point than 12‐ or 16‐week time point (Tukey: *p* < 0.05). In contrast, the amount of orts collected from each cage was impacted by genotype (*p* = 0.047), diet (*p* < 0.001), and week (*p* < 0.001; Table S8). Tukey tests showed no significant differences between genotype, but mice on the doxy diet produced more orts than mice on the control diet (Figure 3B; Figure S3) and more orts were collected at week 16 than week 12.

The percent of active time in which ARBs were observed was impacted by sex (P = 0.002), diet (*p* = 0.027), and genotype (*p* = 0.035; Table S8). Females and mice on the doxy diet performed more ARBs than males and mice on the control diet, but post hoc tests did not show a difference between genotypes (Figure 3C).

### 3.2 Tau concentration and its influence on 16‐week outcome measures

#### 3.2.1 Tau concentration

**FIGURE 4:**
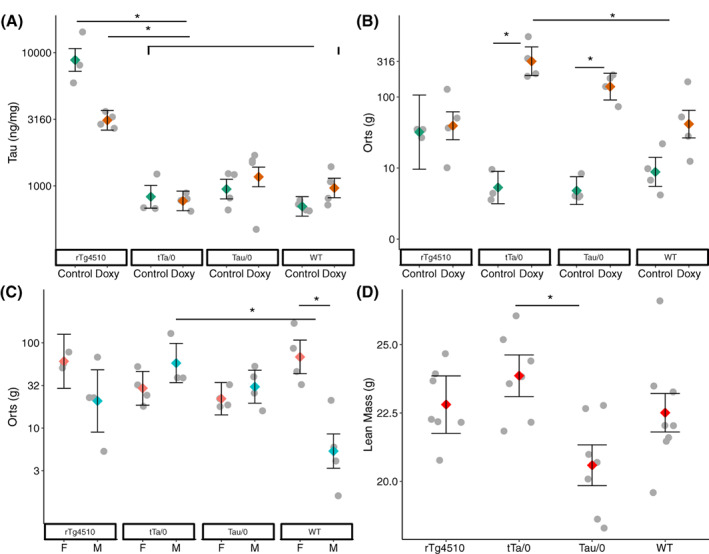
Data from only the 16‐week time point in a mouse model of AD. (A) Tau concentration was significantly impacted by the genotype*diet interaction (adj R^2^ = 0.84, *N* = 30). The amount of orts produced was dependent on (B) genotype*diet and (C) genotype*sex interactions (adj R^2^ = 0.80, N = 30). (D) Lean mass was influenced by genotype (adj R^2^ = 0.32, N = 29). All factor level LSM ± SE are presented over a scatter of individual data point residual values. Y axes in A, B, and C are presented on a log10 back transformed scale. Significant differences in post hoc Significant differences from Tukey tests are depicted by *

At the 16‐week time point, tau concentration in brain tissue was significantly impacted by the genotype*diet interaction (GLM: F_3,21_ = 5.96, η^2^ = 0.460, *p* = 0.004; Figure 4A). rTg4510 mutants on the control diet had higher tau levels than mutants on the doxy diet. Mutants, regardless of diet, had higher tau levels than all other mice (Tukey: *p* < 0.05).

#### 3.2.2 Outcome measures–16‐week time point data

Test statistics for tau on each measure are presented in Table 3, while effects from the full model are listed in Table S10. The concentration of tau only had a significant, negative effect on the proportion of time sleeping (*p* = 0.046, η^2^ = 0.243) and nest scores (*p* = 0.011, η^2^ = 0.234; Table 3) at the 16‐week time point. The proportion of time spent sleeping at week 16 was affected by the sex*genotype (P = 0.023) and sex*diet interaction (*p* < 0.001; Table S10). No differences were found within genotype in terms of proportion of time slept (Tukey: *p* > 0.05), but male mice on the control diet slept for more time than female mice on the control diet and male mice on the doxy diet (Tukey: *p* < 0.05). Mean bout length was only impacted by sex (*p* = 0.043; Table S10): males had longer sleep bouts than females. Nest scores were also impacted by location: mice had higher scores in their home cage than the sleep apparatus (*p* = 0.002; Table S10).

**TABLE 3:** Test statistics from GLMs examining the effect of tau concentration on each 16‐week measure. Bold values represent that the measure was significantly impacted by tau concentration

Proportion of time sleeping	Mean sleep bout length	Nest score^a^	Time at feeder	Total orts	Time spent performing abnormal behaviors^b^	Bone mineral density	Fat mass	Lean mass
F_1,15_ = 4.73	F_1,19_ = 0.80	F_1,25_ = 7.63	F_1,23_ = 0.43	F_1,17_ = 1.34	R^2^ = 0.27	F_1,22_ = 1.90	F_1,11_ = 1.05	F_1,21_ = 0.03
η^2^ = 0.240	η^2^ = 0.040	η^2^ = 0.234	η^2^ = 0.015	η^2^ = 0.073		η^2^ = 0.080	η^2^ = 0.088	η^2^ = 0.001
***p* = 0.046**	*p* = 0.383	***p* = 0.011**	*p* = 0.519	*p* = 0.263		*p* = 0.182	*p* = 0.326	*p* = 0.872

^a^ Genotype was excluded from the model due to collinearity issues with tau concentration.

^b^ Correlation coefficient calculated for tau concentration; data violated GLM assumptions.

Time at the feeder and ARBs in the home cage was not affected by any treatment (*p*'s > 0.05), while the level of orts in the cage was impacted by diet*genotype (*p* = 0.008) and sex*genotype (*p* = 0.005; Table S10) interactions. Differences within genotype or diet are highlighted here, with full pairwise contrasts shown in Table S11. tTa/0 and Tau/0 mice on the doxy diet produced more orts than those on the control diet; tTa/0 mice on the doxy diet produced more orts than WT mice on the doxy diet (Tukey: *p* < 0.05; Figure 4B); female WT and male tTa/0 mice produced more orts than male WT mice (Tukey: *p* < 0.05; Figure 4C).

BMD was impacted by the sex*diet interaction (*p* = 0.012; Table S10), but Tukey tests showed no group differences (*p* > 0.05). Genotype impacted lean mass (P = 0.034; Table S10): tTa/0 mice had higher lean mass than Tau/0 mice (Tukey: *p* < 0.05; Figure 4D). Lean mass also had a positive relationship with the proportion of time active at week 16 (*p* = 0.042; Table S10). Fat mass was impacted by genotype (*p* = 0.010; Table S10), the interaction of sex*genotype*diet (*p* = 0.047), and had a negative relationship with proportion of time active (*p* = 0.002). The only within diet difference is highlighted here, with full pairwise differences in Table S12: male tTa/0 mice on the doxy diet had higher fat mass than male Tau/0 mice on the doxy diet (Tukey: *p* < 0.05).

## 4 DISCUSSION

This study is the first report of several home cage behavioral measures in the rTg4510 mouse model of Alzheimer's disease. While this model reflects the primary pathology seen in AD patients,^34^, ^35^, ^36^, ^37^, ^38^ this study's aim was to determine if more diverse and nuanced behavioral symptoms could be detected. These measures allow the mice to remain in the home cage and be handled less, and therefore reduce human imposed variability into the data.^12^

Tau concentration in brain tissue varied as expected, with rTg4510 mutant mice expressing more tau than other genotypes, and the highest levels present were in those assigned the control diet. However, tau levels only significantly predicted the proportion of time slept and nest scores at the 16‐week time point. The observed negative relationship indicates that mice with more tauopathy sleep for less time and build lower quality nests than those with lower tau levels. This could reflect sleep disruption and decreased ability to perform daily tasks which are commonly seen in human AD patients.^2^ This also confirms past work in this tauopathy model: rTg4510 mutants previously built poorer nests than controls, albeit with very little material,^27^, ^30^ which has previously been shown to affect nest complexity.^40^

Over time, these data show that rTg4510 mutants do display some similar behavior symptoms as human AD patients. Human patients have progressive difficulty performing several tasks involved in daily functioning,^2^ which was reflected in this model based on nest complexity scores. Over time, nest complexity significantly decreased in rTg4510 mutants, which supports previous work in rTg4510 and APP/PS1 models^27^, ^28^, ^29^, ^30^ and likely reflects progressive tauopathy as mice age. However, this cannot be directly concluded since tau was only measured at a single time point. Female rTg4510 mice also had lower nest scores than female WT and Tau/0 mice. This could be due to the higher level of ARB performed by some female rTg4510 mice, discussed below, causing more trampled nests. Finally, nest scores were also impacted by diet over time: mice had less complex nests the longer they received the doxy diet. In humans, case reports have linked doxycycline treatment to extreme levels of anxiety.^51^ While this effect has not been seen in mice, previous work was done on a C57BL/6 background and may not apply to all strains.^52^, ^53^ Indirect evidence shows a relationship between lower quality nests and anxiety measures^54^, ^55^ and in humans, general anxiety can inhibit task performance.^56^, ^57^ Since doxycycline is a common transgene activator, future work should explore the behavioral effects of the drug alone in a variety of strains. However, the effect of diet was primarily due to an extremely low nest score at the end of the study from an rTg4510 mouse on the doxy diet. If this observation were excluded, diet would not have a significant impact. However, the authors could not find a justifiable reason for excluding this data point (no records of flooding or any other abnormal occurrence) and the corresponding cage performed an extreme amount of stereotypic behavior, discussed below.

In contrast to nest scores, most of the measures in this study were not significantly different between mutants and controls. rTg4510 mice did not show any variation in sleep pattern compared to controls. This was surprising since past work in 5XFAD, APP/PS1, and Fus1 KO models show that mutants display more sleep disturbances than controls.^22^, ^23^, ^24^ A larger sample size might reveal differences in sleep patterns, but due to availability, additional mice could not be obtained and our estimation of sample size, using Mead's equation, should have provided ample power (all error terms were greater than 15 degrees of freedom). In terms of odor discrimination, most mice displayed the expected patterns across trial numbers. Neither rTg4510 nor WT mice differed in latency to approach or total time sniffing the odor across trials. This was also surprising since olfactory dysfunction has been recorded in Tg2576 and Tα1‐3RT models.^20^, ^21^ Perhaps this was due to a change in research staff that occurred between the 8‐week and 12‐week trials. Although, mice can have an extreme stress response to odors from males compared to females,^58^ all personnel involved in this study were female. However, the mice could have detected individual differences in staff, which has been previously shown to be a significant factor in data variance.^59^

Although sleep data from the piezoelectric apparatus were not impacted by genotype, general home cage activity varied based on the interaction of sex*diet*genotype. Female rTg4510 mice, on the doxy diet, were more active in the home cage compared to males on the doxy diet. These different patterns may be due to the testing arena. Mice were given a full 24 h to acclimate to the sleep apparatus, but their floor space was greatly reduced from approximately 219cm^2^ to 94.5cm^2^, which could have impacted general activity. rTg4510 mice are known to display age‐dependent hyperactivity,^39^ but given the analyses used in past work, it was not possible to examine interactions between sex, genotype, and age. This past data was also based on infrared sensors, so it was not possible to determine whether hyperactivity was due to abnormal behaviors that develop from poor welfare. Here, females, and mice on the doxy diet performed more ARB, but there was such large variation in the data that any differences between genotype were not significant. Unfortunately, due to the analysis used on this data, treatment interactions could not be formally tested. Anecdotally, we can report that female rTg4510 mice on the doxy diet performed an extreme amount of ARB, with more than 50% of their active time spent looping or route tracing at the 16‐week time point. A video example is included in the supplementary information. This may explain the difference in activity level between female and male rTg4510 mice on the doxy diet as well as the nest score differences between diets and across genotypes in female mice. The excessive looping or route tracing behavior likely trampled any nest structure at the time of observation.

In terms of body composition, genotype predicted lean and fat mass, but rTg4510 mutants did not differ from controls. Activity level had a positive and negative relationship with lean and fat mass respectively, so these endpoint measures could potentially indicate if mice are displaying hyperactivity related to the disease.

Unexpectedly, we observed a large amount of ort production by these mice which were primarily explained by diet: mice on the doxy diet produced more orts than those on the control diet. To the best of our knowledge, grinding behavior has never been reported in this model, even when doxycycline was administered in the same dietary form.^35^, ^37^, ^38^, ^39^ Where previously reported, mice had been group‐housed, so ort production here could be due to solitary housing. The time spent at the feeder did not differ between treatments, but since the mice were given 24 h to acclimate to the video booth for natural behavior recording, most of the behavior could have been performed in this window. Alternatively, the doxy diet used here was anecdotally much softer than the control pellets, so it may have simply been more likely to crumble during regular consumption.

One major limitation of this study was the final age of behavior observation in the mice. Mice were sacrificed at 16 weeks of age, which is when tauopathy can be detected in the cortex.^34^ This stopping age was predetermined based on welfare reasons. Frequent users of this model advised that there is a steep health decline after 16 weeks of age. Although we did not observe many alterations in this study, it is possible that more severe pathology is required to see an effect in these home cage measures. Tau tangles do not form in the hippocampus until 5.5 months of age,^34^ which may explain why there were so few behavioral differences between mutant and control mice. Hippocampal atrophy has previously been shown to predict AD patients from those with mild cognitive impairment, but individual variation prevents this measure from being a comprehensive diagnostic.^60^ In rTg4510 mice specifically, spatial memory and learning deficits increase over time as tauopathy worsens.^35^ Perhaps measurement at a later age would have shown behavioral differences in these mice. For example, over the study period there were no genotype differences in proportion of time slept, but tauopathy at 16 weeks weakly predicted this measure. Extending the testing period may have revealed significant differences between genotypes. Nonetheless, we still expected to see measure differences as time progressed. The 8‐week timepoint was chosen as a baseline measure for all mice, before tauopathy is detectable. Since there is detectable tauopathy, reduced brain mass, and spatial memory deficits by 16 weeks,^35^ behavior changes were still expected in mutants compared to controls at this final timepoint.

Additionally, providing the mice with enrichment items could have altered behavioral measures. Since animals needed to be singly housed to measure most behaviors, we felt it was an ethical necessity to provide more than the average amount of environmental enrichment (10 g nesting material, tube, and chew sticks). This large amount of nesting material likely caused the high nest scores seen across all mice since they had more building materials. Past work provided enrichment in the form of increased cage space, multiple tunnels, chew blocks, huts, and large social groups of 10–15 mice, to the Tg2576 model and the enrichment greatly reduced symptom severity, if given before plaque formation occurs.^61^ While beneficial, these provisions were more complex than what the mice received in our study. However, to the best of our knowledge, enrichment effects have not been empirically evaluated in models of tauopathy. It is possible that enrichment items better represent the stimulating environment that human patients experience, and consequently decreased behavioral differences between the rTg4510 and control mice in this study.

Overall, rTg4510 genotype and tau concentration did not alter many mouse home cage measures back translated from human symptoms. Since these measures are impaired in multiple mouse models of AD, it was expected that rTg4510 model would also show deficiencies. However, general differences between mutants and controls were only found in nesting building. When measures of tauopathy were included in data from the end of the study (16 weeks), this only predicted nest building and time slept. This result may not be completely surprising. While this study was being conducted, data were published showing that the disease phenotype in rTg4510 mutants is primarily due to endogenous gene disruption instead of tau overexpression.^62^ Specifically, a side effect of the transgene inserts is that they disrupt six native mouse genes expressed in the forebrain. This endogenous gene disruption contributes to the pathological phenotype seen in rTg4510 mutants.^62^ Based on this data, this side effect does not appear to impact most of the behavioral measures observed in mice up to 16 weeks of age and may not reflect the diversity of symptoms seen in human AD patients.

## CONFLICT OF INTEREST

The authors declare no competing interests.

## AUTHOR CONTRIBUTIONS

Lindsey A. Robbins and Brianna N. Gaskill conceptualized the study. Amanda J. Barabas and Lindsey A. Robbins curated the data. Brianna N. Gaskill supervised the project. All authors performed the formal data analysis. Amanda J. Barabas wrote the original manuscript and created the data visualization. All authors edited and approved of the final copy.

## Supporting information


**Figure S1** Example image of four mice housed in the piezoelectric sleep apparatus.
**Figure S2.** Example setup used for home cage behavior observation.
**Figure S3.** Example of ort buildup from a mouse on the doxy diet, after 48 hours in the Piezo sleep apparatus.

**Video S1** Supporting Information

**Appendix S1**: Supporting Information

## Data Availability

Data are available in the supplementary information.

## References

[1] Forstl H , Kurz A . Clinical features of Alzheimer's disease. Eur Arch Psychiatry Clin Neurosci. 1999;249:288‐290. 10653284 10.1007/s004060050101

[2] Zhao QF , Tan L , Wang HF , et al. The prevalence of neuropsychiatric symptoms in Alzheimer's disease: systematic review and meta‐analysis. J Affect Disord. 2016;190:264‐271. doi:10.1016/j.jad.2015.09.069 26540080

[3] Albers MW , Tabert MH , Devanand DP . Olfactory dysfunction as a predictor of neurodegenerative disease. Curr Neurol Neurosci Rep. 2006;6:379‐386. doi:10.1007/s11910-996-0018-7 16928347

[4] Doty RL . Odor perception in neurodegenerative disease. In: Doty RL , ed. Handbook of Olfaction and Gustation. 2nd ed. New York, NY: Marcel Dekker, Inc; 2003:479‐502.

[5] Webster SJ , Bachstetter AD , Nelson PT , Schmitt FA , Van Eldik LJ . Using mice to model Alzheimer's dementia: an overview of the clinical disease and the preclinical behavioral changes in 10 mouse models. Front Genet. 2014;5:1‐23. 24795750 10.3389/fgene.2014.00088 PMC4005958

[6] D'Hooge R , De Deyn PP . Applications of the Morris water maze in the study of learning and memory. Brain Res Rev. 2001;36:60‐90. doi:10.1016/S0165-0173(01)00067-4 11516773

[7] Harrison FE , Hosseini AH , McDonald MP . Endogenous anxiety and stress responses in water maze and Barnes maze spatial memory tasks. Behav Brain Res. 2009;198:247‐251. doi:10.1016/j.bbr.2008.10.015 18996418 PMC2663577

[8] Balcombe JP , Barnard ND , Sandusky C . Laboratory routines cause animal stress. Contemp Top Lab Anim Sci. 2004;43:42‐51. 15669134

[9] Meijer MK , Sommer R , Spruijt BM , Van Zutphen LFM , Baumans V . Influence of environmental enrichment and handling on the acute stress response in individually housed mice. Lab Anim. 2007;41:161‐173. doi:10.1258/002367707780378168 17430616

[10] Gouveia K , Hurst JL . Reducing mouse anxiety during handling: effect of experience with handling tunnels. PLoS One. 2013;8:1–8. doi:10.1371/journal.pone.0066401 PMC3688777 23840458

[11] Gouveia K , Hurst JL . Optimising reliability of mouse performance in behavioural testing: the major role of non‐aversive handling. Sci Rep. 2017;7:1‐12. 28322308 10.1038/srep44999 PMC5359560

[12] Crabbe JC , Wahlsten D , Dudek BC . Genetics of mouse behavior: interactions with laboratory environment. Science. 1999;284:1670‐1672. doi:10.1126/science.284.5420.1670 10356397

[13] Auchus RJ , Parker KL . The adrenal glands. In: Kovacs WJ , Ojeda SR , eds. Textbook of Endocrine Physiology. Oxford University Press; 2011:346‐380.

[14] Nostramo R , Sabban EL . Stress and Sympathoadrenomedullary mechanisms. In: Russell J, Shipston M, eds. Neuroendocrinology of Stress. John Wiley & Sons, Ltd; 2015;95‐120.

[15] Rowan AN . Refinement of animal research technique and validity of research data. Toxicol Sci. 1990;15:25‐32. doi:10.1093/toxsci/15.1.25 2197144

[16] Khachaturian ZC . 40 Years of Alzheimer's Research Failure: Now What? MedPageToday. 2018; https://www.medpagetoday.com/neurology/alzheimersdisease/75075

[17] Garner JP . The significance of meaning: why do over 90% of behavioral neuroscience results fail to translate to humans, and what can we do to fix it? ILAR J. 2014;55:55‐456. 10.1093/ilar/ilu047 PMC4342719 25541546

[18] De Visser L , Van Den Bos R , Kuurman WW , Kas MJH , Spruijt BM . Novel approach to the behavioural characterization of inbred mice: automated home cage observations. Genes Brain Behav. 2006;5:458‐466. doi:10.1111/j.1601-183X.2005.00181.x 16923150

[19] Hurst JL , West RS . Taming anxiety in laboratory mice. Nat Methods. 2010;7:825‐826. doi:10.1038/nmeth.1500 20835246

[20] Macknin JB , Higuchi M , Lee VMY , Trojanowski JQ , Doty RL . Olfactory dysfunction occurs in transgenic mice overexpressing human τ protein. Brain Res. 2004;1000:174‐178. doi:10.1016/j.brainres.2004.01.047 15053964

[21] Wesson DW , Levy E , Nixon RA , Wilson DA . Olfactory dysfunction correlates with amyloid‐βburden in an alzheimer's disease mouse model. J Neurosci. 2010;30:505‐514. doi:10.1523/JNEUROSCI.4622-09.2010 20071513 PMC2826174

[22] Coronas‐Samano G , Baker KL , Tan WJT , Ivanova AV , Verhagen JV . Fus1 KO mouse as a model of oxidative stress‐mediated sporadic Alzheimer's disease: circadian disruption and long‐term spatial and olfactory memory impairments. Front Aging Neurosci. 2016;8:1‐26. 27895577 10.3389/fnagi.2016.00268 PMC5108791

[23] Onos KD , Uyar A , Keezer KJ , et al. Enhancing face validity of mouse models of Alzheimer's disease with natural genetic variation. PLoS Genet. 2019;15:1‐29. 10.1371/journal.pgen.1008155 PMC6576791 31150388

[24] Sethi M , Joshi SS , Webb RL , et al. Increased fragmentation of sleep‐wake cycles in the 5XFAD mouse model of Alzheimer's disease. Neuroscience. 2015;290:80‐89. doi:10.1016/j.neuroscience.2015.01.035 25637807 PMC4361816

[25] Deacon R . Assessing burrowing, nest construction, and hoarding in mice. J Vis Exp. 2012;1‐10. 10.3791/2607 PMC3369766 22258546

[26] Gaskill BN , Karas AZ , Garner JP , Pritchett‐Corning KR . Nest building as an indicator of health and welfare in laboratory mice. J Vis Exp. 2013;180:51012. 10.3791/51012 PMC4108067 24429701

[27] Craven KM , Kochen WR , Hernandez CM , Flinn JM . Zinc exacerbates tau pathology in a tau mouse model. J Alzheimers Dis. 2018;64:617‐630. doi:10.3233/JAD-180151 29914030

[28] Filali M , Lalonde R , Rivest S . Subchronic memantine administration on spatial learning, exploratory activity, and nest‐building in an APP/PS1 mouse model of Alzheimer's disease. Neuropharmacology. 2011;60:930‐936. doi:10.1016/j.neuropharm.2011.01.035 21281652

[29] Janus C , Flores AY , Xu G , Borchelt DR . Behavioral abnormalities in APPSwe/PS1dE9 mouse model of AD‐like pathology: comparative analysis across multiple behavioral domains. Neurobiol Aging. 2015;36:2519‐2532. doi:10.1016/j.neurobiolaging.2015.05.010 26089165

[30] Lippi SLP , Smith ML , Flinn JM . A novel hAPP/htau mouse model of Alzheimer's disease: inclusion of APP with tau exacerbates behavioral deficits and zinc administration heightens tangle pathology. Front Aging Neurosci. 2018;10:1–22. doi:10.3389/fnagi.2018.00382 30524268 PMC6263092

[31] Dengler‐Crish CM , Ball HC , Lin L , Novak KM , Cooper LN . Evidence of Wnt/β‐catenin alterations in brain and bone of a tauopathy mouse model of Alzheimer's disease. Neurobiol Aging. 2018;67:148‐158. doi:10.1016/j.neurobiolaging.2018.03.021 29660685

[32] Blennow K , De Leon MJ , Zetterberg H . Alzheimer's disease. Lancet. 2006;368:387‐403. doi:10.1016/S0140-6736(06)69113-7 16876668

[33] Brier MR , Gordon B , Friedrichsen K , et al. Tau and Ab imaging, CSF measures, and cognition in Alzheimer's disease. Sci Transl Med. 2016;8:1‐10. 10.1126/scitranslmed.aaf2362 PMC5267531 27169802

[34] Santacruz K , Lewis J , Spires T , et al. Tau suppression in a neurodegenerative mouse model improves memory function. Science. 2005;309:476‐481. doi:10.1126/science.1113694 16020737 PMC1574647

[35] Blackmore T , Meftah S , Murray TK , et al. Tracking progressive pathological and functional decline in the rTg4510 mouse model of tauopathy. Alzheimer's Res Ther. 2017;9:1‐15. 28931441 10.1186/s13195-017-0306-2 PMC5607580

[36] Cook C , Dunmore JH , Murray ME , et al. Severe amygdala dysfunction in a MAPT transgenic mouse model of frontotemporal dementia. Neurobiol Aging. 2014;35:1769‐1777. doi:10.1016/j.neurobiolaging.2013.12.023 24503275 PMC3992979

[37] Ramsden M , Kotilinek L , Forster C , et al. Age‐dependent neurofibrillary tangle formation, neuron loss, and memory impairment in a mouse model of human tauopathy (P301L). J Neurosci. 2005;25:10637‐10647. doi:10.1523/JNEUROSCI.3279-05.2005 16291936 PMC6725849

[38] Wes PD , Easton A , Corradi J , et al. Tau overexpression impacts a neuroinflammation gene expression network perturbed in Alzheimer's disease. PLoS One. 2014;9:24‐31. 10.1371/journal.pone.0106050 PMC4143352 25153994

[39] Jul P , Volbracht C , De Jong IEM , Helboe L , Elvang AB , Pedersen JT . Hyperactivity with agitative‐like behavior in a mouse tauopathy model. J Alzheimers Dis. 2015;49:783‐795. doi:10.3233/JAD-150292 26519432

[40] Gaskill BN , Gordon CJ , Pajor EA , Lucas JR , Davis JK , Garner JP . Heat or insulation: behavioral titration of mouse preference for warmth or access to a nest. PLoS One. 2012;7:1‐11. 10.1371/journal.pone.0032799 PMC3316552 22479340

[41] Gaskill BN , Garner JP . Power to the people: power, negative results, and sample size. J Am Assoc Lab Anim Sci. 2020;59:9‐16. doi:10.30802/AALAS-JAALAS-19-000042 PMC6978577 31852563

[42] Mead R . The Design of Experiments: Statistical Principles for Practical Applications. Cambridge University Press; 1988.

[43] Yaghouby F , Donohue KD , O'Hara BF , Sunderam S . Noninvasive dissection of mouse sleep using a piezoelectric motion sensor. J Neurosci Methods. 2016;259:90‐100. doi:10.1016/j.jneumeth.2015.11.004 26582569 PMC4715949

[44] Lehmkuhl AR , Dirr ER , Fleming SM . Olfactory assays for mouse models of neurodegenerative disease. J Vis Exp. 2014;e51804. 25177842 10.3791/51804 PMC4827975

[45] Mason GJ . Stereotypies: a critical review. Anim Behav. 1991;41:1015‐1037. doi:10.1016/S0003-3472(05)80640-2

[46] Martin PR , Bateson PPG . Measuring Behaviour: an Introductory Guide. Cambridge University Press; 2007.

[47] Reising NC , Day TA , Hole JT , et al. P1‐114: measurement of endogenous mouse tau in cerebrospinal fluid from aged Pdapp mice following treatment with Ab‐lowering compounds. Alzheimers Dement. 2018;14:P314‐P314.

[48] Lakens D , Scheel AM , Isager PM . Equivalence testing for psychological research: a tutorial. Adv Methods Pract Psychol Sci. 2018;1:259‐269. doi:10.1177/2515245918770963

[49] Ambrée O , Touma C , Görtz N , et al. Activity changes and marked stereotypic behavior precede a β pathology in TgCRND8 Alzheimer mice. Neurobiol Aging. 2006;27:955‐964. doi:10.1016/j.neurobiolaging.2005.05.009 15993515

[50] Richter H , Ambrée O , Lewejohann L , et al. Wheel‐running in a transgenic mouse model of Alzheimer's disease: protection or symptom? Behav Brain Res. 2008;190:74‐84. doi:10.1016/j.bbr.2008.02.005 18342378

[51] Atigari OV , Hogan C , Healy D . Doxycycline and suicidality. BMJ Case Rep. 2014;1‐3. 10.1136/bcr-2013-200723 PMC3888527 24347450

[52] Paris JJ , Singh HD , Ganno ML , Jackson P , McLaughlin JP . Anxiety‐like behavior of mice produced by conditional central expression of the HIV‐1 regulatory protein, tat. Psychopharmacology (Berl). 2014;231:2349‐2360. doi:10.1007/s00213-013-3385-1 24352568 PMC4020990

[53] Vaisburd S , Shemer Z , Yeheskel A , Giladi E , Gozes I . Risperidone and NAP protect cognition and normalize gene expression in a schizophrenia mouse model. Sci Rep. 2015;5:1‐12. 10.1038/srep16300 PMC4639790 26553741

[54] Keleher MR , Zaidi R , Patel K , et al. The effect of dietary fat on behavior in mice. J Diabetes Metab Disord. 2018;17:297‐307. doi:10.1007/s40200-018-0373-3 30918865 PMC6405378

[55] Nolte ED , Nolte KA , Du Yan SS . Anxiety and task performance changes in an aging mouse model. Biochem Biophys Res Commun. 2019;514:246‐251. doi:10.1016/j.bbrc.2019.04.049 31029428 PMC9004632

[56] Bowman MA , Cunningham TJ , Levin‐Aspenson HF , et al. Anxious, but not depressive, symptoms are associated with poorer prospective memory performance in healthy college students: preliminary evidence using the tripartite model of anxiety and depression. J Clin Exp Neuropsychol. 2019;41:694‐703. doi:10.1080/13803395.2019.1611741 31084349

[57] Schweiker‐Marra KE , Marra WT . Investigating the effects of prewriting activities on writing performance and anxiety of at‐risk students. Read Psychol. 2000;21:99‐114.

[58] Sorge RE , Martin LJ , Isbester KA , et al. Olfactory exposure to males, including men, causes stress and related analgesia in rodents. Nat Methods. 2014;11:11‐632. 24776635 10.1038/nmeth.2935

[59] Chesler EJ , Wilson SG , Lariviere WR , Rodriguez‐Zas SL , Mogil JS . Identification and ranking of genetic and laboratory environment factors influencing a behavioral trait, thermal nociception, via computational analysis of a large data archive. Neurosci Biobehav Rev. 2002;26:907‐923. doi:10.1016/S0149-7634(02)00103-3 12667496

[60] Jack CR , Shiung MM , Weigang SD , et al. Brain atrophy rates predict subsequent clinical conversion in normal elderly and amnestic MCI. Neurology. 2005;65:1227‐1231. doi:10.1212/01.wnl.0000180958.22678.91 16247049 PMC2753547

[61] Verret L , Krezymon A , Halley H , et al. Transient enriched housing before amyloidosis onset sustains cognitive improvement in Tg2576 mice. Neurobiol Aging. 2013;34:211‐225. doi:10.1016/j.neurobiolaging.2012.05.013 22727275

[62] Gamache J , Benzow K , Forster C , et al. Factors other than hTau overexpression that contribute to tauopathy‐like phenotype in rTg4510 mice. Nat Commun. 2019;10:1‐12. 31171783 10.1038/s41467-019-10428-1 PMC6554306

