# Supplementary material for: Home cage measures of Alzheimer's disease in the rTg4510 mouse model
Source: Genes Brain Behav. 2022 Jan 19;21(2):e12795. doi: 10.1111/gbb.12795 (PMC9744509; doi:10.1111/gbb.12795)
Supplement: Supplementary file 1 — Figure S1 Example image of four mice housed in the piezoelectric sleep apparatus. Figure S2. Example setup used for home cage behavior observation. Figure S3. Example of ort buildup from a mouse on the doxy diet, after 48 hours in the Piezo sleep apparatus. [file GBB-21-e12795-s003.docx]

# Supplementary Figures


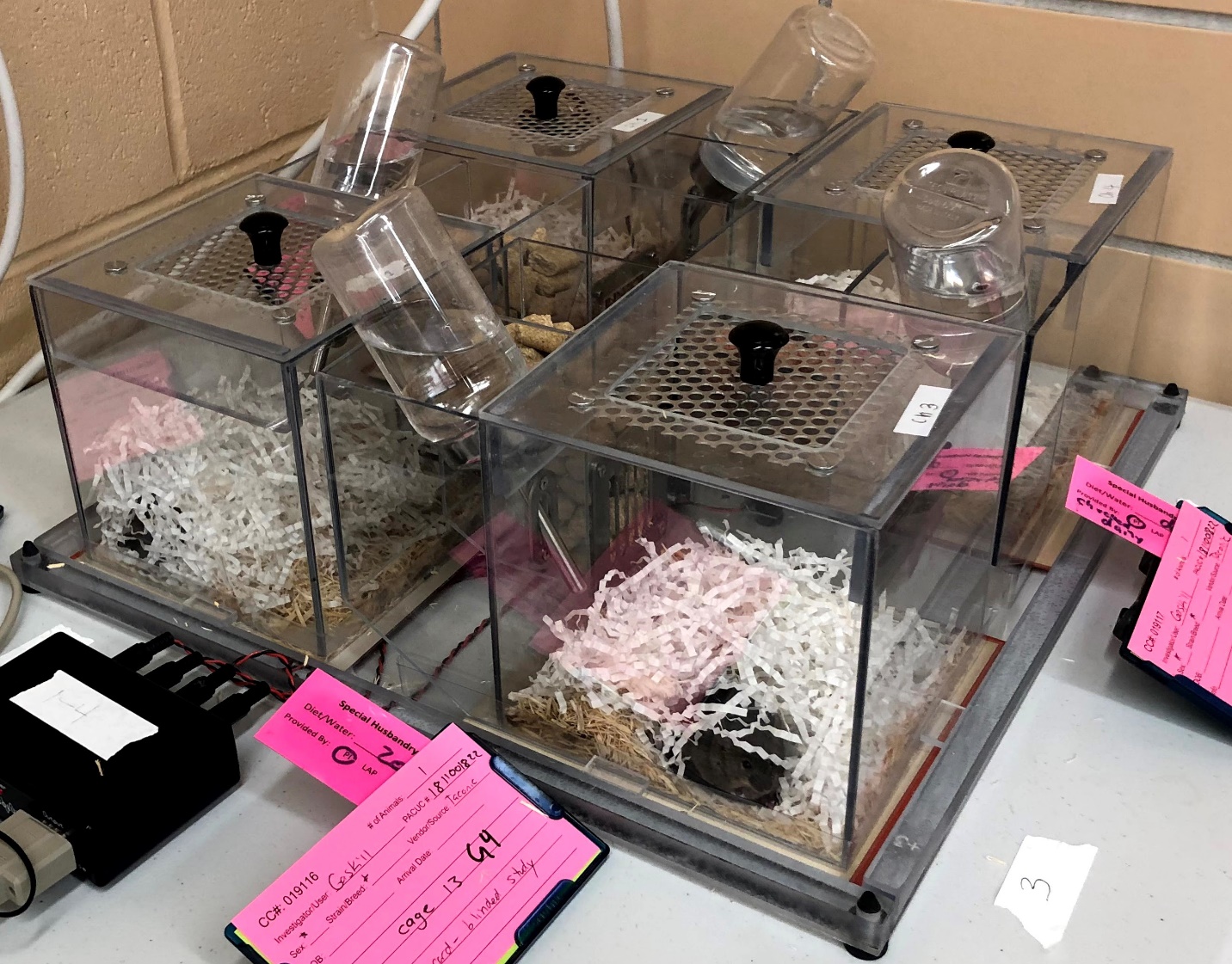


**Fig S1.** Example image of four mice housed in the piezoelectric sleep apparatus.


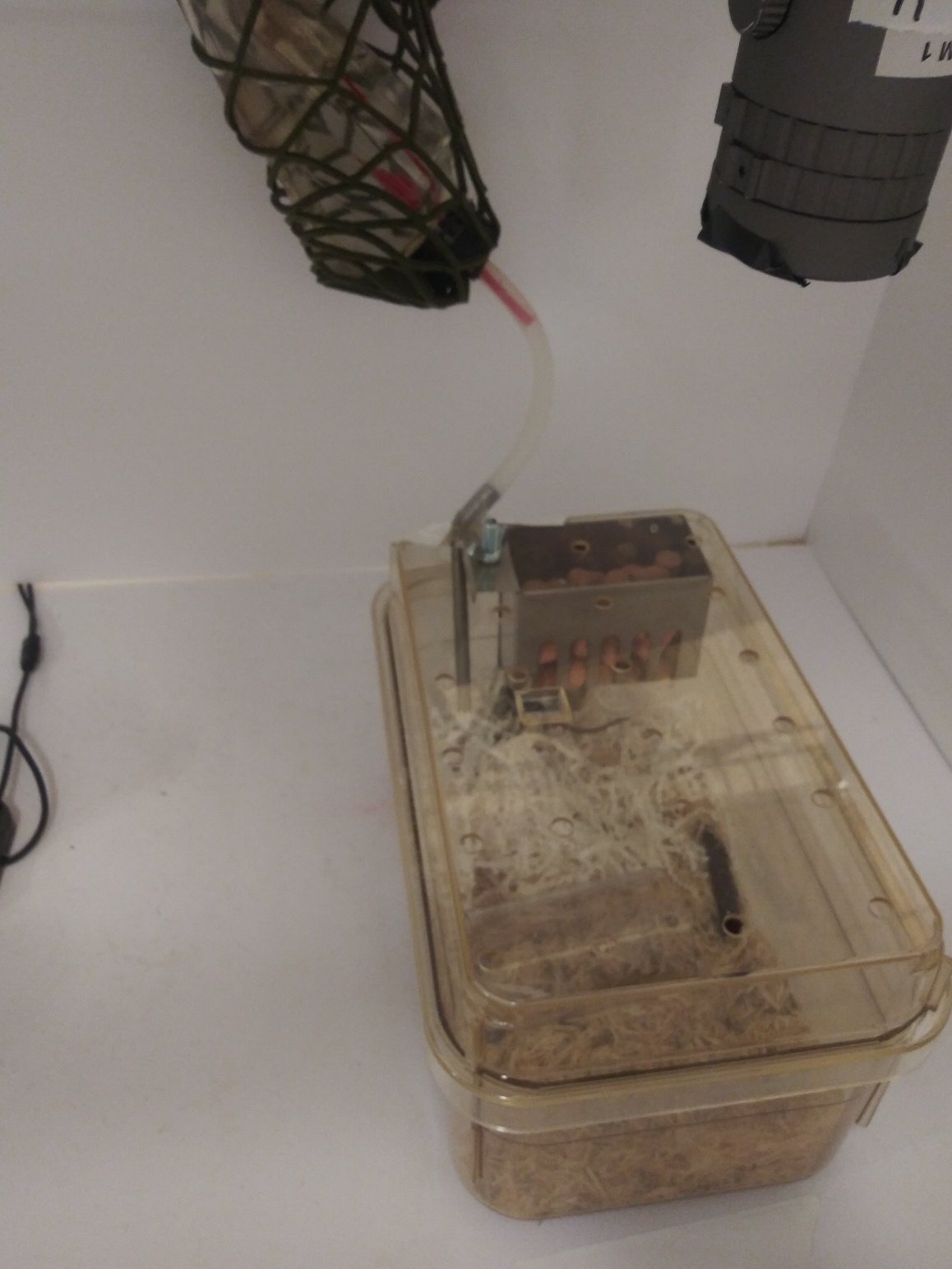


**Fig S2.** Example setup used for home cage behavior observation.


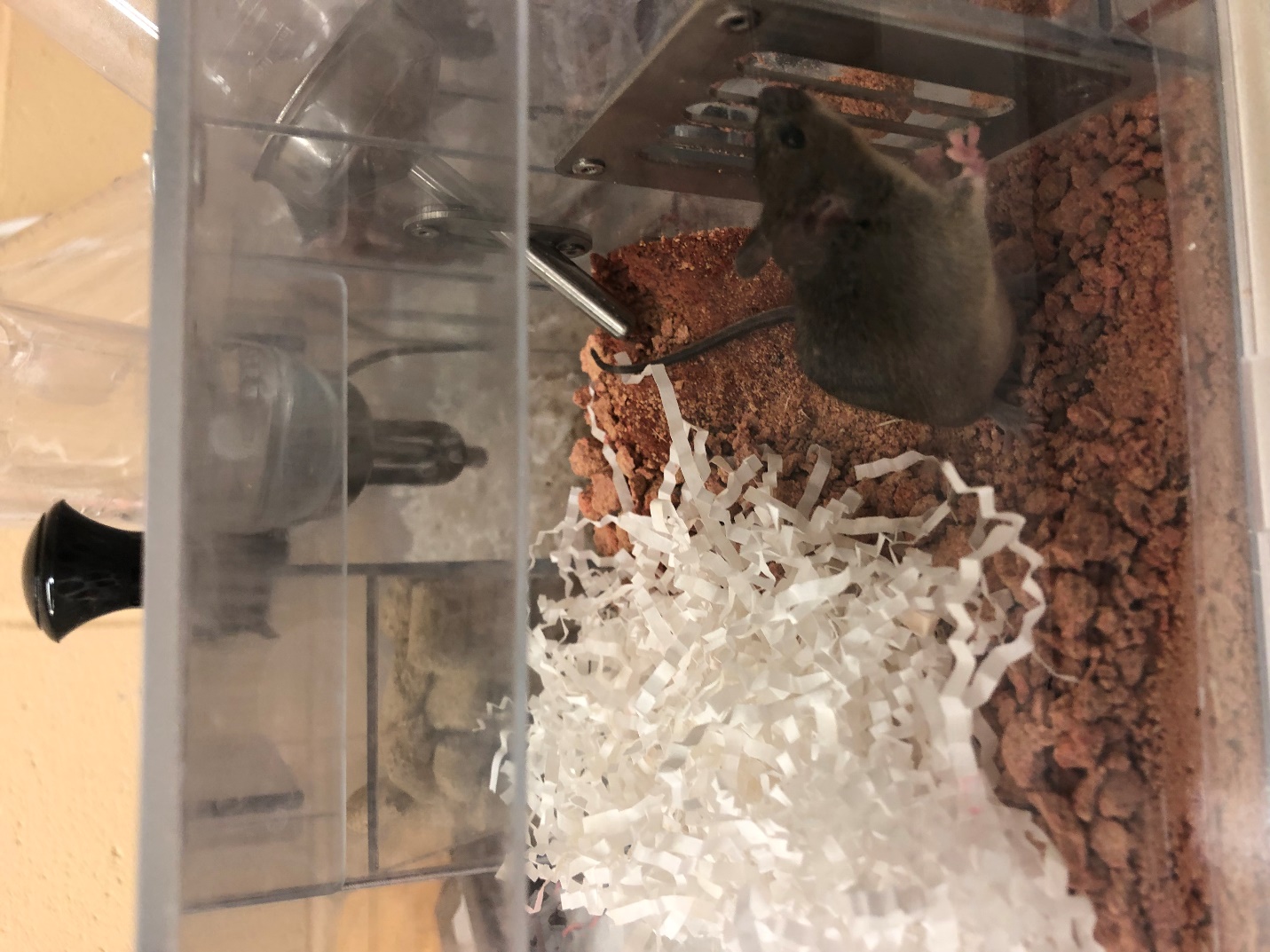


**Fig S3.** Example of ort buildup from a mouse on the doxy diet, after 48 hours in the Piezo sleep apparatus.
